# South African medicinal plant extracts active against influenza A virus

**DOI:** 10.1186/s12906-018-2184-y

**Published:** 2018-03-27

**Authors:** Parvaneh Mehrbod, Muna A. Abdalla, Emmanuel M. Njoya, Aroke S. Ahmed, Fatemeh Fotouhi, Behrokh Farahmand, Dorcas A. Gado, Mansoureh Tabatabaian, Olubunmi G. Fasanmi, Jacobus N. Eloff, Lyndy J. McGaw, Folorunso O. Fasina

**Affiliations:** 1 Department of Veterinary Tropical Diseases, University of Pretoria, Pretoria, South Africa; 2 Influenza and Other Respiratory Viruses Department, Pasteur Institute of IRAN, Tehran, Iran; 3 Phytomedicine Programme, Department of Paraclinical Sciences, University of Pretoria, Pretoria, South Africa; 4 Federal Institute of Industrial Research, Oshodi, Lagos, Nigeria; 5 Department of Production Animal Studies, University of Pretoria, Pretoria, South Africa; 6 Department of Animal Health, Federal College of Animal Health and Production Technology, Ibadan, Nigeria; 7 ECTAD, Food and Agriculture Organization of the United Nations (FAO), Block P, Level 3, United Nations Complex, UN Avenue, Gigiri, Nairobi, Kenya

**Keywords:** Potential medicinal plants, Influenza A virus, Antiviral activity, South Africa, *Rapanea melanophloeos*, *Pittosporum viridiflorum*

## Abstract

**Background:**

Influenza infection remains a major health threat for animals and humans which crucially requires effective antiviral remedies. The usage of herbal medications as readily available alternatives for their compatibility with the body and fewer side effects compared to synthetic chemical treatments has become popular globally. The aim of this study was to investigate and screen in vitro anti-influenza activity of extracts of five South African medicinal plants, namely *Tabernaemontana ventricosa*, *Cussonia spicata*, *Rapanea melanophloeos*, *Pittosporum viridiflorum* and *Clerodendrum glabrum*, species which are used traditionally for the treatment of several diseases such as inflammatory and respiratory diseases.

**Methods:**

Methanol, ethanol (100% and 30%), acetone, hot and cold water extracts of the powdered plants leaves were obtained by standard methods. The cytotoxicity was determined by the MTT colorimetric assay on MDCK cells. The concentrations below CC_50_ values were tested for antiviral activity against influenza A virus (IAV) in different combination treatments. The effect of extracts on viral surface glycoproteins and viral titer were tested by HI and HA virological assays, respectively.

**Results:**

Based on the applied methods, the most effective results against IAV were obtained from *Rapanea melanophloeos* methanol leaf extract (EC_50_ = 113.3 μg/ml) and *Pittosporum viridiflorum* methanol, 100% and 30% ethanol and acetone leaf extracts (EC_50_ values = 3.6, 3.4, 19.2, 82.3 μg/ml, respectively) in all types of combined treatments especially in pre- and post-penetration combined treatments with highly significant effects against viral titer (*P* ≤ 0.01).

**Conclusion:**

The outcomes offer for the first time a scientific basis for the use of extracts of *Rapanea melanophloeos* and *Pittosporum viridiflorum* against IAV. It is worth focusing on the isolation and identification of effective active compounds and elucidating the mechanism of action from these species. However, *Tabernaemontana ventricosa*, *Cussonia spicata* and *Clerodendrum glabrum* leaf extracts were ineffective in vitro in this study.

**Electronic supplementary material:**

The online version of this article (10.1186/s12906-018-2184-y) contains supplementary material, which is available to authorized users.

## Background

Three types of influenza viruses - A, B and C - are classified under the family Orthomyxoviridae. Types A and B are considered to be predominant causes of human and animal infections [1]. However, influenza A virus causes the most severe respiratory diseases which result in considerable morbidity and sometimes high mortality [2]. The common unfavorable symptoms of flu which start normally one to two days after infection are chills, high fever, watering and sore eyes, and rash [3, 4]. Influenza epidemics and pandemics have occurred in the past. The development of vaccines and conventional antiviral agents and their effectiveness against influenza viruses has been limited. Moreover, side effects of antiviral resistance during therapy may affect the central nervous system and the gastrointestinal tract, and have been disappointing mainly due to antigenic shifts and drifts of the virus [5]. Therefore, influenza infections still remain a major cause of mortality and morbidity in humans and animals [6].

Oseltamivir and zanamivir are recommended for both treatment and prophylaxis of influenza to prevent the release of viral particles by blocking neuraminidase. Amantadine and rimantadine have also been used against influenza to inhibit penetration/un-coating of the virus by inhibiting ion-channel M2 protein [7–9]. Clinical efficacy of all these drugs is ambiguous [10] and several cases of drug resistance have been reported [7]. It is well known that medicinal plants have been used all over the world, even in regions with advanced healthcare systems [11], and numerous traditional cultures still rely on indigenous medicinal plants for their primary health care needs [12]. Medicinal plants form an integral part of primary health care in different parts of the world [13–16], and are becoming increasingly popular in modern society as natural alternatives to synthetic medicines [17]. It is possible that the active medicinal plants do not necessarily contain antimicrobial agents, but act by stimulating the victim’s immune system. Traditional herbs are generally cheaper, accessible or readily available and more culturally acceptable. Furthermore, some synthetic drugs cause undesirable side effects [17, 18]. Consequently, a research focus on traditional herbs as complementary therapies or preventive medicine is becoming more attractive.

South Africa is a country which has a strong history of traditional healing, and hosts a wide biodiversity of approximately 30,000 flowering plant species [19], accounting for almost 10% of the world’s higher plant species [20]. Phytochemical studies of medicinal plants have revealed different classes of natural compounds with several pharmacological properties. The various uses of plants include treatment of numerous diseases and life-threatening conditions such as viral diseases and cancer [21]. Medicinal plants have been used to treat influenza disease for decades [1, 22–25]. In our endeavor to discover active plants against IVA, plants were selected based on published traditional indications and pharmacological properties against various diseases and infections. Examples are the bark of *Tabernaemontana ventricosa* tree, which is used to palliate fever and treat wounds and sore eyes. Moreover its wood is suitable for planks and insect-proof material [26]. The ethanolic and aqueous leaf extracts of *Clerodendrum glabrum* have been tested to treat inflammatory diseases [27], and they have anthelmintic, anti-amoebic and antibacterial activities [28]. *Cussonia spicata* bark, roots and leaf extracts have been used for treatment of wounds and they had antibacterial, anti-inflammatory [29], in vitro anthelmintic [30], and in vitro antiplasmodial activities [31]. The leaf and bark extracts of *Pittosporum viridiflorum* had antioxidant activity in HIV-infected patients and could serve as free radical inhibitors [32]. *Rapanea melanophloeos* bark, fruit and leaves are used traditionally for different diseases such as disorders of the stomach, nervous system and respiratory troubles [33]. To the best of our knowledge, the South African medicinal plants under study have never been studied for antiviral activity against influenza A virus. The overall objective of this study was therefore to evaluate and determine the potential activity of the crude extracts of these five medicinal plants against influenza A virus (IAV) infection using the hemagglutination (HA) assay and hemagglutination inhibition (HI) assays.

## Methods

### Preparation of plant extracts

Plants were collected from the Pretoria National Botanical Garden (NBG), South Africa in the summer months. One of the authors (Lyndy J. McGaw), a botanist identified the plant material and voucher specimens were deposited in the HGWJ Schweickerdt Herbarium (PRU), University of Pretoria, South Africa. The identities of the plants were confirmed by herbarium staff. The leaves of plants were air-dried in the shade at room temperature in a well-ventilated room before grinding to fine powder using a Macsalab mill (Model 200 LAB) Eriez, Bramley. Separate aliquots of ground material were extracted (10 ml:1 v:m) using methanol, 30% and 100% ethanol, acetone and water (hot at 40°C, and cold). Each extract was dried under low temperature before reconstituting the extracts at 100 mg/ml in DMSO. The extracts were numbered and stored in a cool environment. The extracts numbers were numbered as follows: 1: *Pittosporum viridiflorum*, aqueous hot, 2: *Pittosporum viridiflorum*, aqueous cold, 3: *Pittosporum viridiflorum*, acetone, 4: *Pittosporum viridiflorum*, 30% ethanol, 5: *Pittosporum viridiflorum*, 100% ethanol, 6: *Pittosporum viridiflorum*, methanol, 7: *Cussonia spicata,* aqueous hot, 8: *Cussonia spicata*, aqueous cold, 9: *Cussonia spicata*, acetone, 10: *Cussonia spicata*, 30% ethanol, 11: *Cussonia spicata*, 100% ethanol, 12: Cussonia spicata, methanol, 13: *Rapanea melanophloeos*, methanol, 14: *Tabernaemontana ventricosa*, methanol and 15: *Clerodendrum glabrum*, methanol.

### Cell culture and influenza virus propagation

Madin Darby Canine Kidney (MDCK) cells purchased from ATCC (CCL-34™) were grown in Dulbecco’s Modified Eagle’s Medium (DMEM) (Mediatech Cellgro, USA), supplemented with 10% Fetal Bovine Serum (FBS) (PAA, Austria) and 1% Pen/Strep (Mediatech Cellgro, USA) at 37 °C in a humidified incubator. The influenza vaccine strain, A/Puerto Rico/8/1934 (H1N1) (ATCC VR-1469™) was obtained from the Influenza Department, Pasteur Institute of Iran. It was propagated in MDCK cells. DMEM supplemented with 1 μg/ml of Trypsin-TPCK (Tosylamide Phenylethyl Chloromethyl Keton-treated Trypsin) (Sigma, USA) without FBS was used as maintenance medium during antiviral experiments. Cell culture infectious dose 50 (CCID_50_) in combination with the hemagglutination assay were used to measure the virus infectivity dose [34, 35].

### Cytotoxicity assay

MDCK cells were incubated in 96-well microplates (Nunc, Denmark) (3 × 10^4^ cell/well) for 24 h at 37 °C. Serial two-fold dilutions of the extracts in complete medium were added to the semi-confluent cells in triplicate and incubated for 48 h. The colorimetric MTT assay was performed according to Mosmann [36] modified by Mehrbod et al. [37]. Briefly, the culture medium was removed and MTT 1X [3-(4,5-dimethyl-2-thiazolyl)-2,5-diphenyl-2H-tetrazolium bromide; Sigma, USA] in 100 μl in PBS was added to each well. Following 3–4 h incubation at 37 °C, the medium containing MTT was removed and DMSO (100 μl) was added to each well to dissolve the formazan crystals to release purple formazan color. The absorbance of the color in the solution was obtained at 570 nm with a microplate reader (BioTek EL 800, US) to calculate viability of the cells using the following formula: (mean Optical Density (OD) of treated cells/mean OD of control cells) × 100. The 50% cytotoxic concentration (CC_50_) which causes visible morphological changes in 50% of the cells with respect to the control cell and effective concentration (EC_50_) which is the concentration required to achieve 50% protection against virus induced cytopathic effect were also calculated using MTT data analyzing by SPSS software. The cells without extract exposure served as negative controls. DMSO as a vehicle control with maximum 0.5% concentration was tested as well.

### Selectivity index

The relative safety of the extracts was obtained by calculating the selectivity index (SI). It is calculated by dividing CC_50_ by EC_50_ in the same units. Selectivity index values higher than 3 indicate potentially safe antiviral activity of the extract [38].

### Antiviral assay

During antiviral evaluations, media supplemented with FBS was removed and the cells were washed with PBS then treated as needed. Influenza virus (100TCID_50_/0.1 ml) was added to the cells in different combination treatments. It was mixed with the extracts EC_50_ for 30 min, then added to the cells and incubated for 1 h at 37 °C (co-penetration procedure). In two other ways, the virus was added to the cells after and/or before the extracts in the span of 1 h (pre-penetration and post-penetration procedures). Following 1 h incubation, unabsorbed viruses were washed and TPCK-containing medium (1 μg/ml) was added. Following 48 h incubation at 37 °C, viabilities of the cells were evaluated by MTT assay as described earlier. Concurrently, the virus titer was determined by testing the cell supernatants using the HA assay [37]. Amantadine hydrochloride (98.5 μg/ml) and oseltamivir carboxylate (394.25 μg/ml) (Sigma, Saint Louis, Missouri, USA) were tested in parallel as control antiviral groups. The cells without extract exposure served as negative controls. DMSO as a vehicle control with maximum 0.5% concentration was tested as well.

### Cellular percentage of protection

The percentage of protection of extracts was calculated using SPSS from the MTT results of mock-infected and infected cells after 48 h exposure, by using the following formula: Percentage of protection = [*(*ODT*)* V − *(*ODC*)* V] / [*(*ODC*)*M − *(*ODC*)* V] × 100 where (ODT)V, (ODC)V and (ODC)M represent the absorbance of the treated sample, the virus-infected control (no compound) and the negative control (mock), respectively [39].

### Hemagglutination assay (HA)

HA activity can be visualized upon mixing virus dilutions with washed chicken erythrocytes in microtitre plates. To evaluate the presence of the virus in cell culture, in either treated or non-treated cells, double serial dilutions of the culture media were added to U-bottom 96-well microplates. Washed chicken red blood cells (cRBCs) (0.5%) were added to each well. The assay was carried out as described previously by Hirst [40] and modified by Mehrbod et al. [37]. HA units were calculated as the reciprocal of the highest dilution giving complete agglutination. Precipitation of the RBCs demonstrates the absence of the virus while hemagglutination and diffuse lattice formation indicates the presence of the virus.

### Hemagglutination inhibition assay (HI)

The lowest amounts of virus particles that can agglutinate the chicken erythrocytes (4HA unit) were used to investigate the inhibitory effect of extracts on the hemagglutinating activity. Briefly, the extracts were serially diluted 2-fold with a concentration range of 10–0.04 mg/ml (25 μl/well). From the virus stock 4HA unit was prepared and added to all wells (25 μl/well). After pre-incubation of 45 min at room temperature, chicken erythrocytes (50 μl/well) were added to the solution and after 1 h the physical interaction between extracts and virus surface HA glycoprotein was read by the agglutination inhibition pattern.

### Phytochemical pre-screening

Qualitative phytochemical pre-screening was performed to allow the detection of potentially interesting compounds at the earliest stages of separation. A spot of the crude extracts was developed on TLC plates and separately eluted using CEF [Chloroform: Ethyl acetate: Formic acid (90:10:1)] and EMW [Ethyl acetate: Methanol: Water (40:4.5:4)] solvent systems [41]. The zones on the developed TLC plates were visualized under UV light (254 and 365 nm) and further localized by exposure to a vanillin-sulphuric acid spray reagent.

### Statistical analysis

The data expressed as mean ± SD was analyzed by one-way analysis of variance (ANOVA) and General Linear Model (GLM) (SPSS 18.0) LSD and Duncan post-hoc tests. Sample values with *p* ≤ 0.05 and *p* ≤ 0.01 were considered statistically significant and highly significant, respectively.

## Results

In this study, methanol, ethanol, acetone and hot and cold aqueous extracts of five selected plant species were tested for their antiviral efficacy against IAV. The cytotoxicity of the extracts was tested to determine the non-toxic concentrations for cell viability prior to antiviral assay. With different extracts and virus combined treatments, the antiviral activity varied with the different extracts of the same plant as determined by the HA assay. The profile of the five medicinal plants used in this study is listed in Table 1.

**Table 1:** Profile of the five medicinal plants used in this study

Botanical name	Family	Local Name	Plant part used	Voucher number
*Tabernaemontana ventricosa* Hochst. ex. A.DC.	Apocynaceae	Bospaddaboom	leaf	PRU 120680
*Cussonia spicata* Thunb.	Araliaceae	Kiepersol	leaf	PRU 115683
*Rapanea melanophloeos* (L.) Mez	Myrsinaceae	Kaapseboekenhout, boekenhout	leaf	PRU 120670
*Pittosporum viridiflorum* Sims	Pittosporaceae	Kasuur	leaf	PRU 120025
*Clerodendrum glabrum* E.Mey. var. *glabrum*	Verbenaceae	Tontelhout	leaf	PRU 114809

### Cytotoxicity of plant extracts on MDCK cells and their selectivity indices against virus

In this study, the acetone, methanol, ethanol, hot and cold aqueous extracts of five selected medicinal plants were differentially cytotoxic to MDCK cells and the appropriate concentrations were determined for cell viability and further assays. The medicinal plant *Rapanea melanophloeos* had the highest CC_50_ value of 227 ± 13.6 μg/ml followed by *Clerodendrum glabrum* with 221 ± 34.9 μg/ml. The lowest CC_50_ value (0.1 ± 0.07 μg/ml) was obtained with *Tabernaemontana ventricosa* (Table 2). The results for CC_50_ were broadly different for different extracts (Table 2). The EC_50_ of the extracts were calculated from the MTT results by one-way ANOVA analysis and compared to the negative control with no significant effects on cell viability. These results were obtained from the extract concentrations that reduced viral titer and maintenance of cell viability (Table 2). The selectivity index values of the extracts were calculated with the highest SI value of 8 obtained with *Cussonia spicata* and lowest SI value of 2 obtained with *Pittosporum viridiflorum*, *Rapanea melanophloeos*, *Tabernaemontana ventricosa* and *Clerodendrum glabrum*. The CC_50_ values for Amantadine hydrochloride and oseltamivir carboxylate in MDCK cells were calculated as 197 μg/ml and 788 μg/ml, respectively. Concentrations of 1510.0 mg/ml and 3120.0 mg/ ml were used as EC_50_ of amantadine and oseltamivir, respectively.

**Table 2:** CC_50_, EC_50_ and SI of the extracts

Plant name and number^a^	Extract type	CC_50_ (μg/ml) (mean ± SD)	EC_50_ (μg/ml)	SI
*Pittosporum viridiflorum, 2*	Cold water	129 ± 12.6	32.2	4
*Pittosporum viridiflorum, 3*	Acetone	165 ± 25.2	82.3	2
*Pittosporum viridiflorum, 4*	30% ethanol	77 ± 24.8	19.2	4
*Pittosporum viridiflorum, 5*	100% ethanol	7 ± 5.8	3.4	2
*Pittosporum viridiflorum, 6*	Methanol	15 ± 9.3	3.6	4
*Cussonia spicata, 9*	Acetone	108 ± 2.4	13.5	8
*Cussonia spicata, 11*	100% ethanol	39 ± 12.6	4.8	8
*Cussonia spicata, 12*	Methanol	117 ± 11.5	14.6	8
*Rapanea melanophloeos, 13*	Methanol	227 ± 13.6	113.3	2
*Tabernaemontana ventricosa, 14*	Methanol	0.1 ± 0.07	0.05	2
*Clerodendrum glabrum, 15*	Methanol	221 ± 34.9	110.4	2

*CC*_*50*_ 50% cytotoxic concentration, *EC*_*50*_ 50% effective concentration, *SI* Selectivity Index

^a^Number refers to the plants numbering throughout the manuscript

*2: Cold water extract of Pittosporum viridiflorum; 3: Acetone extracts of Pittosporum viridiflorum; 4: 30% ethanolic extract of Pittosporum viridiflorum;; 5: 100% ethanolic extract of Pittosporum viridiflorum; 6: Methanolic extract of Pittosporum viridiflorum; 9: Acetone extract of Cussonia spicata; 11: 100% ethanolic extract of Cussonia spicata; 12: Methanolic extract of Cussonia spicata; 13: Methanolic extract of Rapanea melanophloeos; 14: Methanolic extract of Tabernaemontana ventricosa; 15: Methanolic extract of Clerodendrum glabrum.*

### Inhibitory effect of plant extracts on influenza A virus

**Fig. 1:**
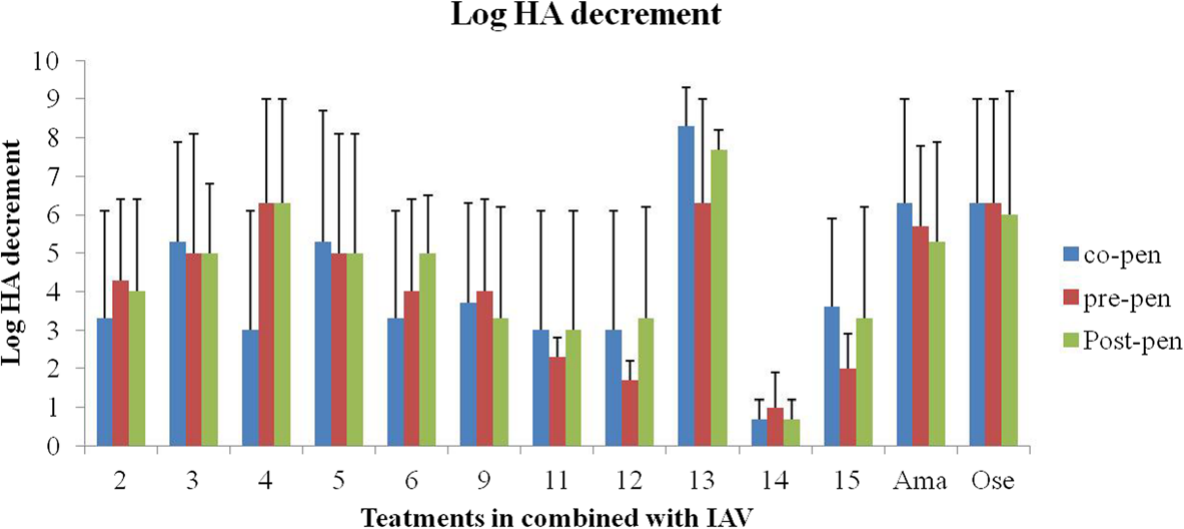
Log_10_ HA decrement obtained from HA assay. Data presented as mean ± SD are averages of 3 independent HA titrations. Ama: Amantadine and Ose: Oseltamivir. The negative controls have no virus added and remained at zero value through-out the study

Different crude extracts were tested in an in vitro micro-inhibition screening assay to determine antiviral activity against influenza A virus. Antiviral activity of the crude extracts of the selected plants was analyzed based on the Log_10_ HA titer (Table 3) and Log HA decrement (Fig. 1). Extracts from two species *Pittosporum viridiflorum* and *Rapanea melanophloeos,* had remarkable and promising antiviral activity against IAV. The averages of 7.4 and 5 logs HA decrements were observed in all types of combined treatments of *Rapanea melanophloeos* and *Pittosporum viridiflorum*, respectively. Log_10_ HA titer from HA assay in combined treatments showed that these two plant extracts applied the most significant activity on the virus titer especially in pre- and post-penetration procedures for *Pittosporum viridiflorum,* but in all types of combined treatments for *Rapanea melanophloeos*.

**Table 3:** Log_10_ HA titer from HA assay in combined treatments with virus compared to virus control group

	Treatment	Extract type	Log HA (mean ± SD)		
			Co-pen	Pre-pen	Post-pen
Combined treatments	*Pittosporum viridiflorum, 2*	Cold water	1.51 ± 1.17	1.20 ± 0.93**	1.30 ± 1.02*
	*Pittosporum viridiflorum, 3*	Acetone	0.90 ± 0.97**	1.00 ± 1.12**	1.00 ± 0.82**
	*Pittosporum viridiflorum, 4*	30% ethanol	1.61 ± 1.24	0.60 ± 0.93**	0.60 ± 0.93**
	*Pittosporum viridiflorum, 5*	100% ethanol	0.90 ± 1.17**	1.00 ± 1.12**	1.00 ± 1.12**
	*Pittosporum viridiflorum, 6*	Methanol	1.51 ± 1.17	1.30 ± 1.02*	1.00 ± 0.78**
	*Cussonia spicata, 9*	Acetone	1.40 ± 1.09	1.30 ± 1.02*	1.51 ± 1.17
	*Cussonia spicata, 11*	100% ethanol	1.61 ± 1.24	1.81 ± 0.47	1.61 ± 1.24
	*Cussonia spicata, 12*	Methanol	1.61 ± 1.24	2.01 ± 0.41	1.61 ± 1.02
	*Rapanea melanophloeos, 13*	Methanol	0.00 ± 0.00**	0.60 ± 0.93**	0.30 ± 0.00**
	*Tabernaemontana ventricosa, 14*	Methanol	2.31 ± 0.16	2.21 ± 0.16	2.31 ± 0.16
	*Clerodendrum glabrum, 15*	Methanol	1.40 ± 0.78	1.91 ± 0.56	1.51 ± 1.17
	Amantadine hydrochloride	–	0.60 ± 0.93**	0.80 ± 0.82**	0.90 ± 0.97**
	Oseltamivir carboxylate	–	0.60 ± 0.93**	0.60 ± 0.93**	0.70 ± 1.09**
No extract treatment	Influenza virus	–	2.51 ± 0.31	2.51 ± 0.31	2.51 ± 0.31

Data presented as mean ± SD are averages of 3 independent HA titration. *, **: Significantly and highly significantly different from values obtained for extract-treated samples compared to virus inoculated untreated sample (*P* ≤ 0.05 & *P* ≤ 0.01) analyzed by SPSS, LSD post-hoc test

### Cell viability in combined treatments

The results of the MTT assay for antiviral activity of the extracts against IAV revealed that treatments exposed to different types of combined treatments of extracts EC_50_ and virus 100 CCID_50_ had highly significant increment in ODs compared to the virus-inoculated cells alone (*P* ≤ 0.01). Exposure of the cells to amantadine and oseltamivir EC_50_ combined with the virus resulted in high cell viability (*P* ≤ 0.01) (Table 4).

**Table 4:** Cell viabilities from MTT assay in combined treatments with virus compared to virus control group

Treatment	Extract type	Cell viability (mean ± SD)		
		Co-pen	Pre-pen	Post-pen
*Pittosporum viridiflorum, 2*	Cold water	0.59 ± 0.11**	0.70 ± 0.18**	0.65 ± 0.15**
*Pittosporum viridiflorum, 3*	Acetone	0.85 ± 0.17**	0.85 ± 0.23**	0.84 ± 0.18**
*Pittosporum viridiflorum, 4*	30% ethanol	0.57 ± 0.06**	0.79 ± 0.11**	0.70 ± 0.16**
*Pittosporum viridiflorum, 5*	100% ethanol	0.54 ± 0.07**	0.56 ± 0.10**	0.48 ± 0.07**
*Pittosporum viridiflorum, 6*	Methanol	0.49 ± 0.07**	0.76 ± 0.10**	0.74 ± 0.16**
*Cussonia spicata, 9*	Acetone	0.79 ± 0.11**	0.90 ± 0.09**	0.76 ± 0.10**
*Cussonia spicata, 11*	100% ethanol	0.59 ± 0.05**	0.81 ± 0.16**	0.57 ± 0.10**
*Cussonia spicata, 12*	Methanol	0.53 ± 0.04**	0.55 ± 0.21**	0.56 ± 0.14**
*Rapanea melanophloeos, 13*	Methanol	0.75 ± 0.23**	0.83 ± 0.16**	0.80 ± 0.23**
*Tabernaemontana ventricosa, 14*	Methanol	0.60 ± 0.12**	0.63 ± 0.21**	0.67 ± 0.17**
*Clerodendrum glabrum, 15*	Methanol	0.71 ± 0.25**	0.80 ± 0.20**	0.78 ± 0.25**
Amantadine hydrochloride	–	0.79 ± 0.13**	0.75 ± 0.09**	0.71 ± 0.14**
Oseltamivir carboxylate	–	0.83 ± 0.16**	0.83 ± 0.18**	0.69 ± 0.14**
Influenza virus	–	0.20 ± 0.05	0.20 ± 0.05	0.20 ± 0.05

Data presented as mean ± SD are averages of 4 independent MTT assays. **: highly significantly different from values obtained for drugs-treated samples compared to untreated sample (*P* ≤ 0.01) analyzed by SPSS, LSD post-hoc test

### Cellular percentage of protection

The ODs were also analyzed to examine the percentage of extract protection on cell viability against the virus infectivity assay in combined treatments. Data are recorded in Table 5. Extract numbers 3, 9, 13 and 15 had the highest consistent percentage of protection in all types of combined treatments.

**Table 5:** Cellular percentage of protection in combined treatments with virus compared to control groups

Treatment	Extract type	Percentage of protection (mean ± SD)		
		Co-pen	Pre-pen	Post-pen
*Pittosporum viridiflorum, 2*	Cold water	49.26 ± 10.78^ab^	64.19 ± 28.68^bcd^	56.61 ± 20.33^bc^
*Pittosporum viridiflorum, 3*	Acetone	81.71 ± 14.37^e^	81.69 ± 23.00^de^	79.67 ± 13.33^d^
*Pittosporum viridiflorum, 4*	30% ethanol	47.29 ± 9.44^ab^	75.09 ± 17.48^cde^	63.84 ± 25.29^bcd^
*Pittosporum viridiflorum, 5*	100% ethanol	43.97 ± 18.11^a^	46.29 ± 12.70^ab^	35.60 ± 14.63^a^
*Pittosporum viridiflorum, 6*	Methanol	38.27 ± 18.56^a^	71.45 ± 18.57^cde^	68.54 ± 22.62^cd^
*Cussonia spicata, 9*	Acetone	74.87 ± 13.14^de^	88.45 ± 17.11^e^	70.34 ± 6.59^cd^
*Cussonia spicata, 11*	100% ethanol	50.50 ± 18.12^ab^	76.68 ± 12.20^de^	47.22 ± 6.28^ab^
*Cussonia spicata, 12*	Methanol	41.94 ± 10.49^a^	42.54 ± 17.52^a^	45.69 ± 12.00^ab^
*Rapanea melanophloeos, 13*	Methanol	68.75 ± 18.78^cde^	78.75 ± 9.03^de^	74.39 ± 19.81^cd^
*Tabernaemontana ventricosa, 14*	Methanol	53.13 ± 26.48^abc^	55.90 ± 30.94^abc^	59.65 ± 22.68^bcd^
*Clerodendrum glabrum, 15*	Methanol	63.63 ± 19.51^bcd^	74.22 ± 15.10^cde^	71.87 ± 22.00^cd^
Amantadine hydrochloride	–	73.94 ± 9.94^de^	69.88 ± 10.02^cde^	64.50 ± 10.29^bcd^
Oseltamivir carboxylate	–	79.44 ± 9.89^de^	79.26 ± 12.85^de^	61.37 ± 11.71^bcd^

Data presented as mean ± SD are averages of 4 independent tests. Different letters show significant differences in each column (Duncan Grouping)

### Hemagglutination-inhibition

The current study indicated that treatment of IAV with the extracts reduced the hemagglutination activity of the virus, which may result from the physical interaction of the extracts with virus hemagglutinin. All plant extracts in different dilutions, even after repeating the assay twice, did not show any inhibition of RBC agglutination. This can be attributed to arguing against physical HA interaction of the extract ingredients with RBC.

### Phytochemical pre-screening

Several different compounds were present in the TLC of the crude extracts based on the different colors with vanillin-sulphuric acid spray reagent. Further purification will be performed to isolate the active constituents.

## Discussion

In the current report antiviral activities of some traditionally used medicinal plants were investigated for inhibitory effects against influenza A virus infection. Previous reports have defined quality standards for antiviral evaluation: a stringent endpoint of EC_50_ values less than 100 μg/ml, including SI values of more than 3 are the defined standard for antiviral efficacy of natural products [38, 42]. Medicinal plants have progressively been explored as suitable alternative sources of antiviral agents [1, 43–45] and research efforts continue. After applying the different treatments and evaluation of the viral HA titer, all extracts were highly significantly protective on the cell viability in all types of combined treatments (*P* ≤ 0.01). The methanol extract of *Rapanea melanophloeos* and different extracts of *Pittosporum viridiflorum* afforded the most effective HA results against IAV (*P* ≤ 0.01). The crude extracts of *Cussonia spicata*, *Tabernaemontana ventricosa* and *Clerodendrum glabrum,* which had good SI values (more than 3), were weakly active against IAV with no significant decrease in IAV HA titer (*P* ≥ 0.05). Amantadine hydrochloride and oseltamivir carboxylate displayed a significant eliminating effect on the HA titer in comparison with the extracts of *Rapanea melanophloeos* and *Pittosporum viridiflorum* and they were better than most other extracts tested in this study. Regarding the log_10_ HA decrement, the most effective extracts at the first level were extracts of *Rapanea melanophloeos* with an average of 7.4 log decrement and *Pittosporum viridiflorum* with an average of 5 log decrements. The lowest effect belonged to *Tabernaemontana ventricosa* methanol extract with 0.8 log decrement. The known conventional antiviral drugs amantadine and oseltamivir are promisingly effective agents, but growing resistance of viruses has created a significant challenge [46, 47] and it is recommended to focus on new alternatives especially those of natural origin. By following the HI assay it was confirmed that none of the extracts showed physical interaction with virus HA surface glycoproteins. Moreover, by GLM analysis of the results, estimated marginal means of all the respective values were calculated for different exposure ways (combined treatment) which confirmed all the outcomes. The data are shown in Additional file 1: Figure S1, Additional file 2: Figure S2, Additional file 3: Figure S3 and Additional file 4: Figure S4.

Several studies have demonstrated that influenza infections lead to uncontrolled elevations of pro-inflammatory cytokines, making this infection a strong risk factor for severe complications which could be terminal [48–51]. Therefore, effective alternative therapeutics to conventional antiviral drugs can be based on anti-inflammatory and immunomodulatory agents [52]. The ethanolic and aqueous extracts of *Clerodendrum glabrum* have been tested for efficacy in treating inflammatory diseases [27]. Additionally, the leaf extract of this plant exhibited anthelmintic, anti-amoebic and antibacterial activity [28]. The leaf extract of *Clerodendrum glabrum,* which is high in flavonoid content, is used for cough, cold, sore throat and chest complaints. The local Zulu people in South Africa take leaf infusions for cough and fever [53, 54]. Leaf infusions are also taken by the Vhavenda for cold, sore throat, chest complaints and as an insect repellent [54]. Von Koenen [55] reported that the Zulu make an extract of the root and it is suggested that this extract is used to assist fever and cough. It was also reported to have anti-inflammatory, in vitro anthelmintic [30] and other activities against intestinal parasites and diabetes [53, 54], *Plasmodium falciparum* strain D10 [56], Gram-positive and Gram-negative bacteria [57] and fungal pathogens [58]. The medicinal plant *Cussonia spicata* had antiviral activity by causing a 2 log reduction in feline herspesvirus type 1 [59]. Therefore, the above mentioned plant species might be promising alternatives to decrease the unfavorable effects of flu.

Influenza is a viral pathogen that imposes a burden of central nervous system (CNS) disease as well. An increasing incidence of influenza-associated encephalitis has been reported [60, 61]. It is important to notice that oxygen and nitrogen free radicals are also involved in pathogenesis of influenza virus infection [62]. Oxygen radicals and nitric oxide are over-generated in a variety of microbial infections. They cause tissue injury and mutagenesis through oxidation and nitration of various bio-molecules. The above-mentioned scenario may be explained if plants with antioxidant potency possess antiviral activity as well. This is in agreement with the findings of the current study, where the antiviral medicinal plant *Pittosporum viridiflorum* investigated in this study previously had antioxidant and free radical inhibition properties [32]. It also has been used traditionally in the treatment of opportunistic fungal infections in HIV/AIDS patients in the Eastern Cape Province, South Africa [63]. Infusions of *Pittosporum viridiflorum* leaves have been used to treat cryptococcal meningitis in the Eastern Cape Province of South Africa [64]. Moreover, a 2 log reduction in feline herspesvirus type 1 growth was discovered [59].

*Rapanea melanophloeos* is a medicinal plant used by Zulu traditional healers to manage blood-clot related diseases [65]. Various extracts (methanol, n-hexane, chloroform, ethyl acetate and aqueous) prepared from the bark of *Rapanea melanophloeos* were screened for phytochemicals as well as antioxidant and anti-platelet aggregation activity. Phytochemical screening of this plant showed the presence of tannins, terpenoids, alkaloids, saponins, cardiac glycosides, flavonoids and phlobatannins [65]. The traditional use of *Rapanea melanophloeos* by South Africans in curing TB-related symptoms such as fever, cough, chest disease, night sweats etc. has been reported [53]. This plant is traditionally used in Kenya to reduce parasitism in small ruminants [66]. Sakurasaponin was isolated from the methanolic leaf extract of *Rapanea melanophloeos* and was found to be active against *Cladosporium cucumerinum* [67]. However, in the domain of influenza disease management where there are annual epidemic burdens and fatal pandemics from time to time, there has been no study of any of the above-mentioned plants for their effect against IAV so far. In this study, we evaluated the antiviral activity of extracts of these plants against influenza A virus infection. Unfortunately, the extracts 1, 7, 8, and 10 which were obtained from hot and cold aqueous and 30% ethanol of *Pittosporum viridiflorum* and *Cussonia spicata* did not dissolve well in DMSO as the consistent solvent used and they were removed from the experiment.

## Conclusions

Medicinal plants used for treating various disorders may have useful biological activities, particularly against infectious diseases. Crude plant extracts contain a diversity of constituents that may exert their antiviral effect either alone or as synergistic effects of compounds together. In this study, based on the evaluation of antiviral results, only 5 extracts [methanol extract of *Rapanea melanophloeos* and 100% and 30% ethanol, methanol and acetone extracts of *Pittosporum viridiflorum*] were selected for further evaluation. These plants may have the capacity to ease the symptoms of flu. However, no scientific research has been conducted previously to validate the antiviral activity of these plant species. The fact that some degree of inhibition was observed with these extracts may suggest that the extracts contain active component(s) that may be responsible for the observed anti-influenza activity. The next focus of this study will be the isolation and characterization of compounds responsible for bioactivity against IAV infection as well as their mode of action.

## Additional files

Additional file 1: **Figure S1.** Estimated Marginal Means of Log HA titer. This graph shows the Log HA titer levels analyzed by GLM. (TIF 51 kb)

Additional file 2: **Figure S2.** Estimated Marginal Means of Log HA decrement. This graph shows the decrement levels in Log HA titers analyzed by GLM. (TIF 50 kb)

Additional file 3: **Figure S3.** Estimated Marginal Means of cell viability. This graph shows the ODs of the cell viability test analyzed by GLM. (TIF 53 kb)

Additional file 4: **Figure S4.** Estimated Marginal Means of percentage of protection. This graph shows the protection of the extracts on the cell viability analyzed by GLM. (TIF 56 kb)

## Abbreviations

ANOVA: Analysis of variance
CC_50_: 50% cytotoxic concentration
CCID_50_: Cell culture infectious dose 50
CEF: Chloroform: Ethyl acetate: Formic acid
cRBCs: Chicken red blood cells
DMEM: Dulbecco’s Modified Eagle’s Medium
EC_50_: 50% effective concentration
EMW: Ethyl acetate: Methanol: Water
FBS: Fetal Bovine Serum
GLM: General linear model
HA: Hemagglutination assay
HI: Hemagglutination inhibition assay
IAV: Influenza A virus
MDCK: Madin Darby Canine Kidney
MTT: [3-(4,5-dimethyl-2-thiazolyl)-2,5-diphenyl-2H-tetrazolium bromide]
OD: Optical density
SI: Selectivity index
TLC: Thin layer chromatography
Trypsin-TPCK: Tosylamide Phenylethyl Chloromethyl Keton-treated Trypsin
