# Supplementary figures and images for: South African medicinal plant extracts active against influenza A virus
Source: BMC Complement Altern Med. 2018 Mar 27;18:112. doi: 10.1186/s12906-018-2184-y (PMC5872571; doi:10.1186/s12906-018-2184-y)

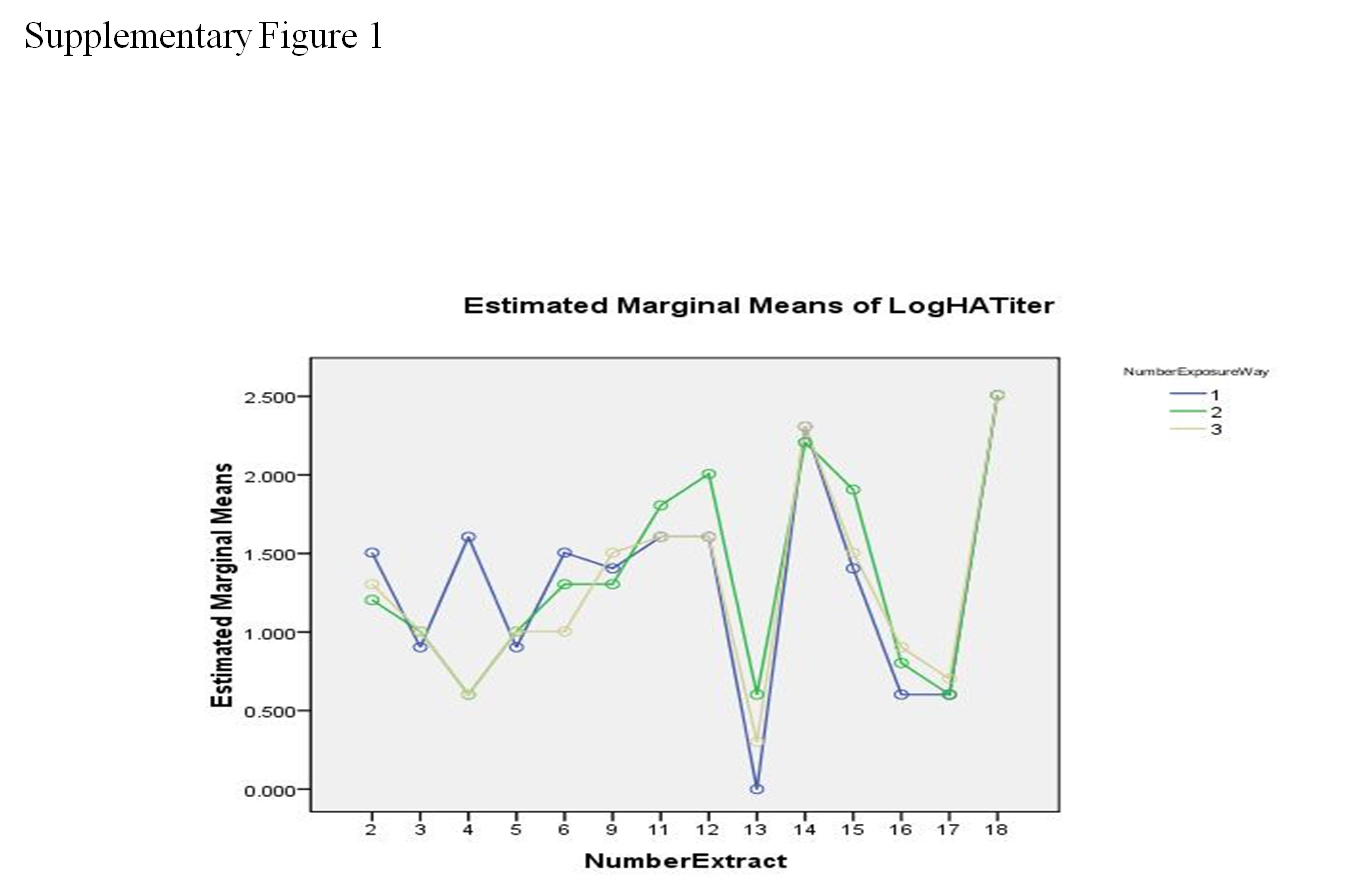

Supplement: Supplementary file 1 — Figure S1. Estimated Marginal Means of Log HA titer. This graph shows the Log HA titer levels analyzed by GLM. (TIF 51 kb) [file 12906_2018_2184_MOESM1_ESM.tif]

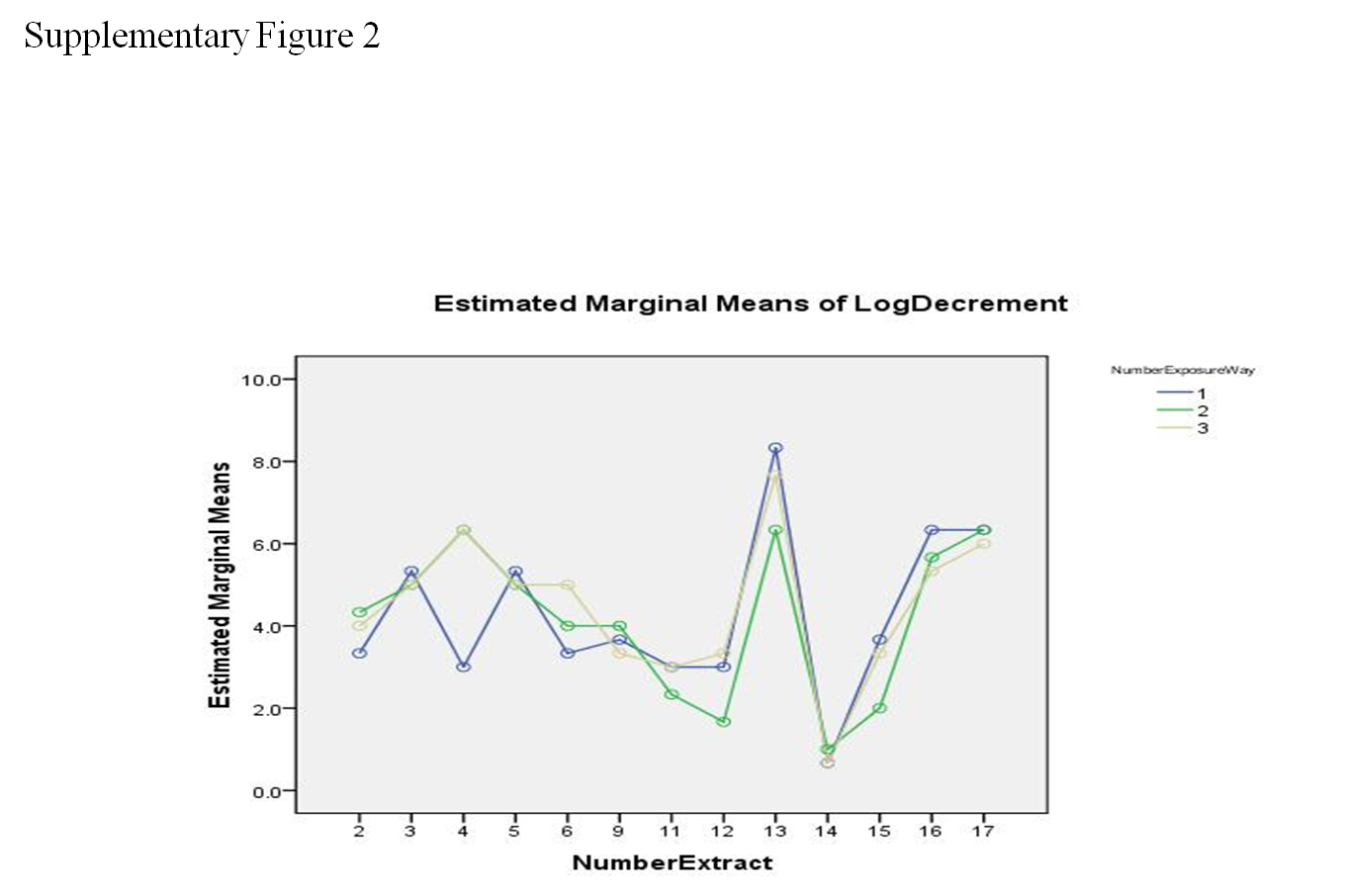

Supplement: Supplementary file 2 — Figure S2. Estimated Marginal Means of Log HA decrement. This graph shows the decrement levels in Log HA titers analyzed by GLM. (TIF 50 kb) [file 12906_2018_2184_MOESM2_ESM.tif]

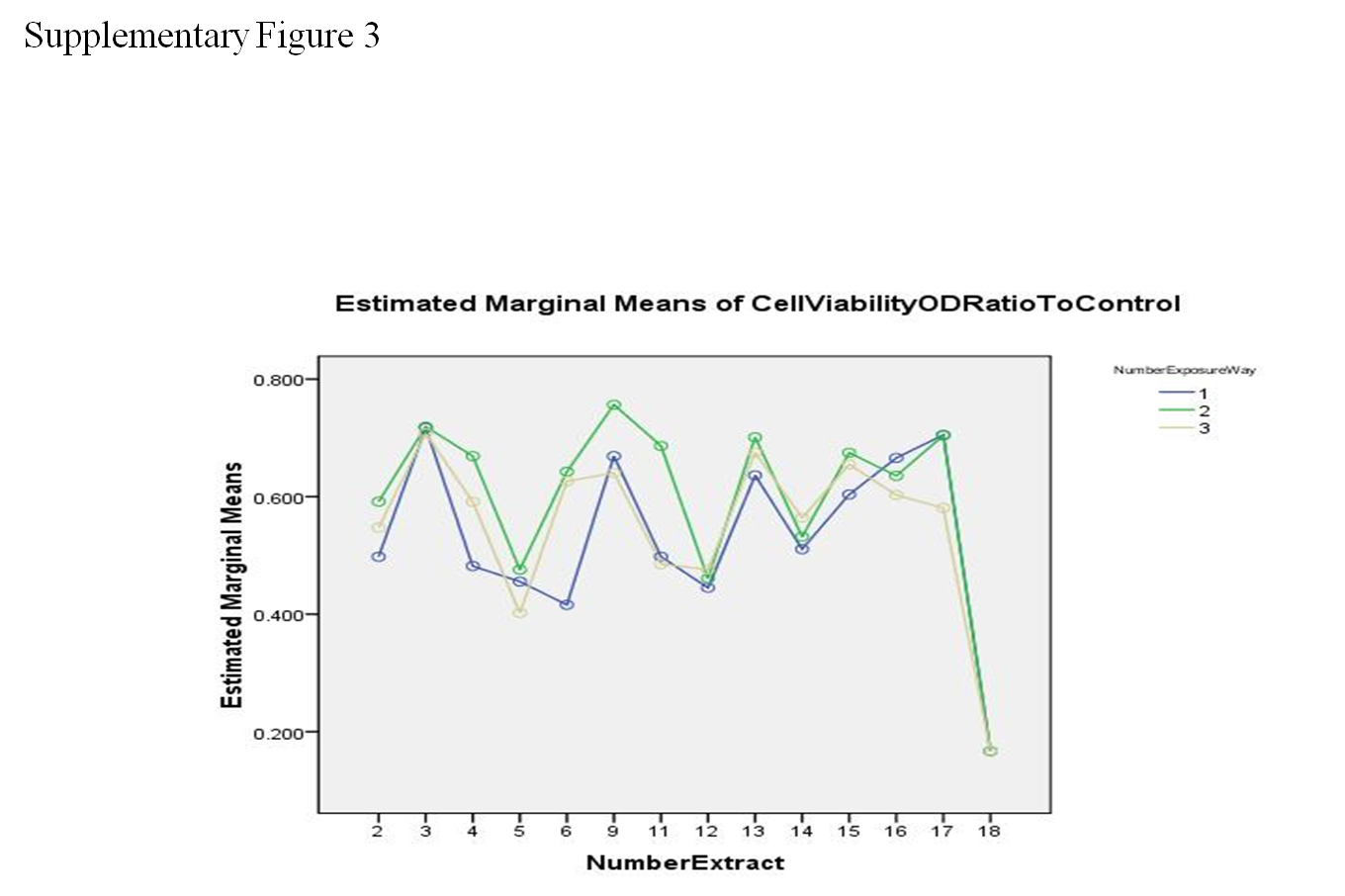

Supplement: Supplementary file 3 — Figure S3. Estimated Marginal Means of cell viability. This graph shows the ODs of the cell viability test analyzed by GLM. (TIF 53 kb) [file 12906_2018_2184_MOESM3_ESM.tif]

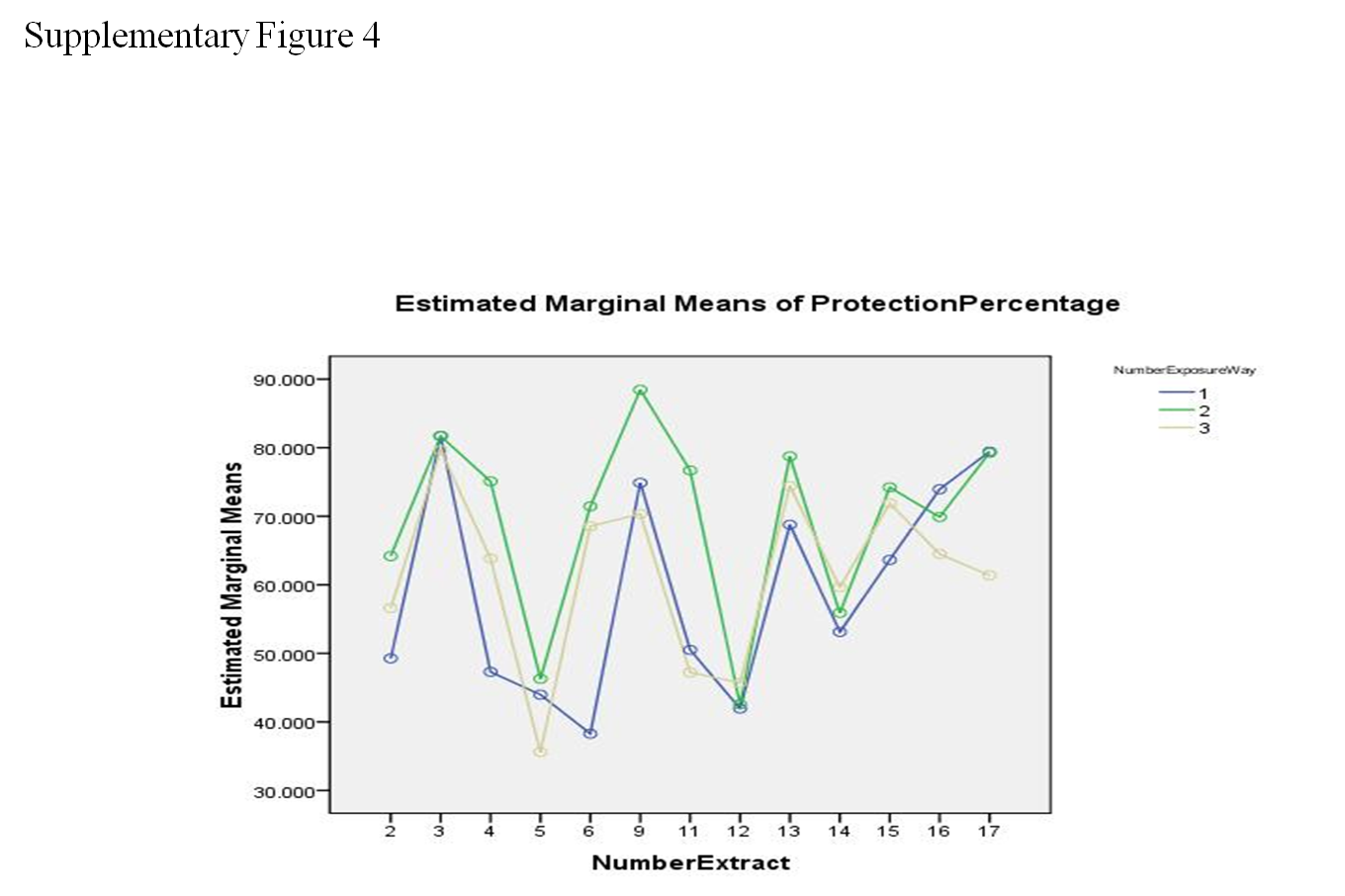

Supplement: Supplementary file 4 — Figure S4. Estimated Marginal Means of percentage of protection. This graph shows the protection of the extracts on the cell viability analyzed by GLM. (TIF 56 kb) [file 12906_2018_2184_MOESM4_ESM.tif]
